# Application of Microfluidics in the Production and Analysis of Food Foams

**DOI:** 10.3390/foods8100476

**Published:** 2019-10-11

**Authors:** Boxin Deng, Jolet de Ruiter, Karin Schroën

**Affiliations:** Food Process Engineering Group, Wageningen University, Bornse Weilanden 9, 6708 WG Wageningen, The Netherlands; jolet.deruiter@wur.nl

**Keywords:** microfluidics, monodispersity, emulsions, foams, up-scaling, dynamic surface tension, coalescence

## Abstract

Emulsifiers play a key role in the stabilization of foam bubbles. In food foams, biopolymers such as proteins are contributing to long-term stability through several effects such as increasing bulk viscosity and the formation of viscoelastic interfaces. Recent studies have identified promising new stabilizers for (food) foams and emulsions, for instance biological particles derived from water-soluble or water-insoluble proteins, (modified) starch as well as chitin. Microfluidic platforms could provide a valuable tool to study foam formation on the single-bubble level, yielding mechanistic insights into the formation and stabilization (as well as destabilization) of foams stabilized by these new stabilizers. Yet, the recent developments in microfluidic technology have mainly focused on emulsions rather than foams. Microfluidic devices have been up-scaled (to some extent) for large-scale emulsion production, and also designed as investigative tools to monitor interfaces at the (sub)millisecond time scale. In this review, we summarize the current state of the art in droplet microfluidics (and, where available, bubble microfluidics), and provide a perspective on the applications for (food) foams. Microfluidic investigations into foam formation and stability are expected to aid in optimization of stabilizer selection and production conditions for food foams, as well as provide a platform for (large-scale) production of monodisperse foams.

## 1. Introduction

**Figure 1:**
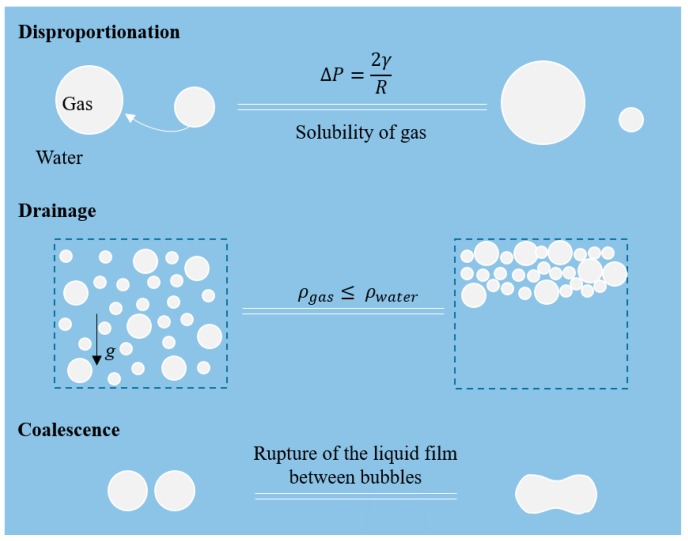
Illustration of the three main destabilization mechanisms of foams, from top to bottom: Disproportionation, drainage, and coalescence.

Foam is a colloidal system consisting of a high-volume fraction of gas phase and a liquid (or solid) phase. It is made by mixing or injecting gas bubbles into the liquid (that later may be solidified). Emulsifiers, if any, in the foaming system are used for stabilizing the newly created gas-water interface by decreasing the surface tension (*γ*), and modifying the interfacial viscoelasticity as well as the repulsive interaction forces between bubbles. However, foams are thermodynamically unstable. The principles of stability have extensively been described by Walstra (1989) [1]. The main destabilization mechanisms of foam are disproportionation (also termed Oswald ripening), drainage, and coalescence, and are illustrated in Figure 1. These mechanisms occur simultaneously, and act synergistically. Disproportionation is the most predominant and fast process. It is a result of the pressure difference between bubbles, due to the Laplace pressure *∆P* = $\frac{2\gamma }{R}$ in each bubble. Since the gas in the small bubbles (small radius *R*) has a higher pressure than that in large bubbles, it tends to diffuse through the liquid phase towards large bubbles. The average bubble size increases with time, with large bubbles growing at the expense of small ones. As a result, polydisperse foams are more prone to coarsening and subsequent destabilization. Disproportionation is especially pronounced in foams due to the high solubility of gas in water—in contrast, the effect is much smaller for emulsions, which consist of liquid droplets dispersed in another immiscible liquid phase (the solubility of e.g., triglyceride is very low in water). During foam drainage, water leaks out of the foam due to gravity (*g*: Acceleration) based on the large density difference between gas phase ($\rho_{gas}$) and water phase ($\rho_{water}$), and the bubbles rise. Their rising velocity scales with R^2^, demonstrating the enhancement of drainage by disproportionation. Furthermore, due to the drainage process, the liquid film between bubbles becomes thinner, which initiates coalescence of bubbles.

In the food industry, foaming is used to make less dense, airy food products, and thus modify their appearance and texture. Bubble stabilization is crucial for this, and occurs in typical examples of liquid food foams: Whipped tops, cappuccino, ice-cream, etc. In these products, proteins (typical dairy proteins) are mostly used as emulsifiers. Synergistic stabilization effects between proteins and other ingredients were also reported, such as for milk foam for cappuccino that is stabilized by proteins and polysaccharides [2]. Traditional foaming operation is carried out by rotor-stator mixing, turbulent mixing, and steam injection. These techniques use energy inefficiently, and the size distribution of generated bubbles is highly polydisperse, which is one of the underlying reasons for food foam instability (by disproportionation) as mentioned above [3].

To retard food foam destabilization, creation of monodisperse foams is a way to go, although this is difficult with conventional foaming techniques. Another way is to prevent coalescence or restrict bubbles rise, for example by absorbing stabilizers to the air–water interface or constructing a network in the continuous phase, respectively. Recently, colloidal particles have been introduced as novel stabilizers that can perform both of these stabilizing functions. The resulting dispersions are termed Pickering foam (or Pickering emulsion), for a review see Lam et al. (2014) [4]. For food applications a variety of food-grade (biobased) particles are studied, such as particles derived from proteins and (or) starch [5,6], fat crystals [7]. These particles can absorb to the air–water interface where they may increase the steric barriers between bubbles, as well as increase the interfacial viscoelasticity, and (or) form a network in the continuous phase and increase its viscosity [4]. The deposition of particles and the exact stabilization mechanisms still need to be detailed.

From the above it is clear that although food foams are made at a large-scale, understanding of the underlying mechanisms could still be improved, and for that microfluidic technology could provide valuable tools to study ‘traditional’ foams, as well as Pickering foams. The main topics that need to be addressed are bubble formation, interface stabilization by emulsifiers or particles, and destabilization processes in foams. It is expected that if these aspects are understood this will lead to better use of ingredients, and thus design of food foams based on first principles.

In the last decades, food research has benefited from a large number of developments in the field of microfluidic technology, in particular for applications in emulsions. Microfluidic droplet formation units are known to produce very small and uniform droplets at low energy consumption albeit that the relatively low throughput limits their applicability. To overcome this restriction up-scaling has been attempted; achievements and associated challenges are discussed in a review by Schroën and colleagues [8]. Besides, microfluidic devices have also been developed as analytical tools to aid in the characterization of emulsions [9,10,11], for example, to measure dynamic interfacial tension in the droplet formation process and to predict the emulsion coalescence stability. For further information, we recommend a review by Gunes (2018) [12] dedicated to studies using microfluidics and complex interfaces as present in foods.

Extensive studies using microfluidic devices have been performed in liquid–liquid systems (emulsions). Yet, the literature on gas–liquid systems (foams) remains comparatively scarce, not to mention literature related to food foams with complex (mixtures of) stabilizers. This is likely related to bubble formation being a much faster process than droplet formation, which is due to the low viscosity of the to-be-dispersed phase. It thus requires extremely short observation time scales, in particular when small micrometer-sized bubbles are needed. Based on the findings obtained from emulsions, microfluidic devices are expectedly highly suited to produce monodisperse (food) foams, and to study foam formation as well as stabilization. In this review, we will focus on reported contributions in the microfluidic field specifically dedicated to foams (where available), and also interpret methods and insights from emulsion studies and relate them to what can be expected for foams taking into account the fast dynamics at (sub)millisecond time scales. We will round off describing how microfluidic devices can be used to produce monodisperse food foams at high throughput.

## 2. Microfluidic Devices

**Figure 2:**
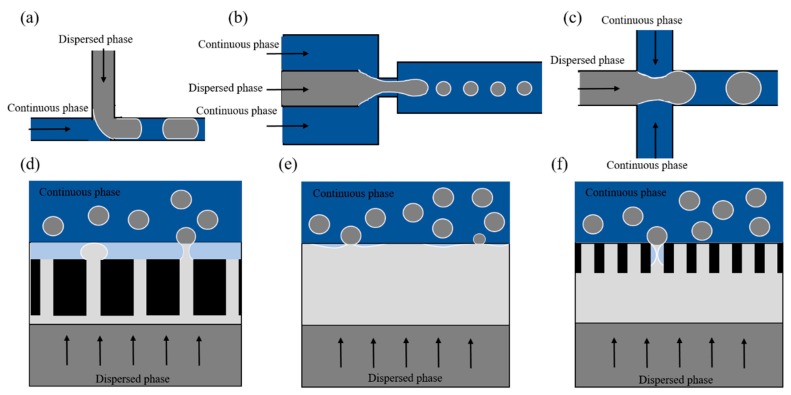
Schematic layout of microfluidic devices: (**a**) T-junction, (**b**) flow-focusing, and (**c**) co-flowing device; (**d**) terrace-based microchannel (MC); (**e**) Edge-based Droplet GEneration (EDGE), and (**f**) partitioned-EDGE droplet formation unit. Please note that in the top three devices channels have the same depth, while in the bottom three, a height difference is needed to allow spontaneous droplet formation to take place. Deep channels (of all the designs) and shallow areas (in the bottom three designs) are indicated as dark grey and light grey, respectively. The continuous phase (water phase) has a blue color, and shows as dark blue when flowing in the deep channel, and as light blue when in the shallow areas. The gas has no color in this figure. The white outline indicates the gas—water interface. These schematics are not drawn to scale.

Various microfluidic devices have been introduced for the formation of monodisperse foams, and they use either a shear force, or spontaneous snap-off for bubble formation. The typical shear-based geometries include T-junction (Figure 2a) [13,14,15,16], flow-focusing (Figure 2b) [17,18,19,20,21], and co-flowing devices (Figure 2c) [22]. On the other hand, devices that use spontaneous snap-off are microchannels (e.g., terrace-based microchannel, which is a so-called 2.5D geometry; Figure 2d) [23,24] and Edge-based Droplet GEneration (EDGE; Figure 2e, also termed STEP) devices [25].

In shear-based devices (also termed active devices), droplet formation is controlled by an external force: The shear stress exerted by the continuous phase flow. In spontaneous droplet formation devices (also termed passive devices), droplet breakup is induced by the minimization of surface energy through a combined effect of specific geometry and surface tension. Both types of microfluidic devices have their pros and cons. An extensive review that describes both types of microfluidic devices in great detail, and also includes membrane emulsification is by Vladisavljevic and co-workers (2012) [26]. Perspectives towards spontaneous emulsification devices for food production have been covered by Maan et al. (2011) [27], who also gave a comparison between spontaneous and shear-based devices [28].

## 3. Bubble and Droplet Formation

In the bubble (and droplet) formation process, various forces are involved to create and stabilize the gas/liquid (and liquid/liquid) interface. The three main forces are the surface tension force, viscous force, and inertial force. One of the key contributions to quantitatively describe bubble (and droplet) formation is to define a set of dimensionless numbers that capture the relative importance of one force over another; thereby defining various formation regimes. When the dimensionless number is around unity, both of its constituting forces influence bubble and droplet formation, whereas a single force dominates at either low or high dimensionless number values. The dimensionless numbers used in this review are defined and summarized in Table 1.

Dominating forces are different for the regimes of spontaneous and shear-based formation, and for bubbles and droplets. Typically, spontaneous formation of bubbles and droplets operates at relatively low to-be-dispersed and continuous phases velocities, leading to negligible effects of viscous stress (e.g., low capillary number—Ca) and inertia force (e.g., low Weber number—We). Surface tension is the dominating force in that case. In shear-based formation, the capillary number of the continuous phase, Ca_c_, is increased through the continuous phase flow velocity—which introduces significant viscous stress during emulsification. Often shear-based formation is required for high-throughput emulsion or foam production, e.g., to sustain monodisperse droplet or bubble formation when the dispersed phase velocity is high. If the dispersed phase is a liquid (e.g., emulsification) its high velocity introduces viscous stresses in the neck of the droplet (increased Ca_d_; low Reynolds number—Re_d_). In the case of gas (e.g., foaming), its velocity is even higher than for a liquid under the same applied pressure. This is expected to introduce inertial stresses in the neck of the bubble (increased We_d_; high Re_d_). To compare various situations, Table 1 is instrumental: The influence of a single process parameter can be derived through these dimensionless number, for instance the influence of the continuous phase viscosity.

### 3.1. Shear-Based Systems

Microfluidic investigations of foam have been carried out mostly with shear-based systems using model components. To translate generated findings to food foams, the bubble size needs to be derived from device geometry and process parameters. Complex scaling relations based on geometrical characteristics have been suggested. In a T-junction (see Figure 2a):

(1) $$R_{\mathrm{pinch}}=w+w_{\mathrm{in}}-(\frac{hw}{h+w}-\varepsilon )+(2(w_{\mathrm{in}}-(\frac{hw}{h+w}-\varepsilon ))(w-(\frac{hw}{h+w}\;-\;\varepsilon ))){}^{1/2},$$

where, *w* is the width of the main channel (filled with continuous phase and formed bubbles), and *w_in_* is the width of the side channel (filled with to-be-dispersed phase); *h* is the channel height, and *ε* the rounded corner of the device. Essentially, this equation uses the radii of the receding interface of the bubble growing at the end of the squeezing process, which is based on the blockage of the main channel by the to-be-dispersed phase to predict the volumetric size of bubbles (and droplets) [29]. The size of bubbles (and droplets) is proportional to the device geometry dimension. Alternatively, simpler relations have been suggested that revolve around a force balance [13], or around basic observations related to the gas–liquid flow rate ratio, $\frac{Q_{gas}}{Q_{liquid}}$ [20,21]. For example, also in the squeezing regime in a T-junction, based on the pressure drop across the forming bubble, Garstecki et al. (2006) [13] found *L = d × (*$\frac{Q_{gas}}{Q_{liquid}}$*) + w* to predict the length (*L*) of plug bubbles (and droplets). Here, *d* is the characteristic width of the neck of the gaseous thread, and *w* the width of the main channel. In a flow-focusing device (see Figure 2b), different scaling relations were found. Within the same formation regime as mentioned above, Fu et al. (2010) [21] scaled *L* with the gas-to-liquid flow rate ratio and liquid Reynolds number as $\frac{L}{W_{c}}=1.40{\left(\frac{Q_{gas}}{Q_{iiquid}}\right)}^{1.10}Re^{0.46}$ (in which *Wc* is the microchannel width), to further incorporate the effect of continuous phase viscosity on the size of slug bubbles; in the dripping regime the normalized bubble diameter (*d_b_*) was described as $\frac{d_{b}}{D}=1.1{\left(\frac{Q_{gas}}{Q_{liquid}}\right)}^{0.4}$, in which *D* is the dimension of the formation orifice [30].

**Figure 3:**
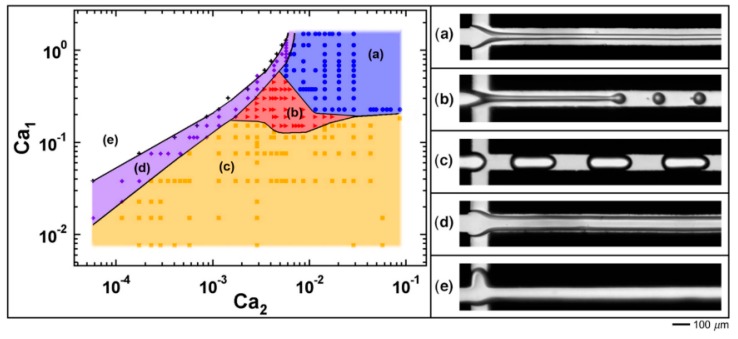
Capillary number-based flow patterns in a flow-focusing microchannel. Ca_1_ and Ca_2_ are the capillary numbers of the to-be-dispersed and continuous phases, respectively. The five flow patterns are: (**a**) Threading; (**b**) jetting; (**c**) dripping; (**d**) tubing; (**e**) and viscous displacement. Reprinted from Cubaud et al. (2008) [31], with the permission of AIP publishing.

Each scaling relation is valid only for the channel geometry and flow conditions they were derived for. Translating these relations to systems that contain other components, or even to other devices is difficult (if not impossible) due to changes in the device geometry (or dimension) and wetting conditions, bubble formation mechanisms, etc. For example, the bubble formation mechanism in T-junctions is dependent on both the device geometry and the process conditions, and varies from dripping, to transition, and eventually jetting. For flow-focusing devices, five flow patterns (see Figure 3) were observed as a function of the capillary numbers Ca of both phases, and scaling relations for droplet size have been developed for the dripping and jetting regimes [31]. For more information on scaling relations, we recommend reviews by Tu and colleagues (2015) [32] and Drenckhan et al. (2015) [33]. They summarize different bubble formation mechanisms and related scaling relations, and, in the latter review, hydrodynamic forces that are involved during bubble formation.

Bubble and droplet formation in microfluidic devices have also been numerically modeled, to complement the experimental scaling laws. An example is the work by Dang et al. (2015) [34] using a Volume of Fluid (VOF) method and, secondly, a comparative method of coupled Level Set and VOF (CLSVOF). Numerical simulations yield valuable insights in the effects of changing device geometry and fluid properties, and can thus be used for engineering design studies to optimize foaming and emulsification processes. Accurate droplet size predictions are obtained particularly in case of geometry-dominated droplet formation regimes. For a more thorough understanding the dynamic nature of the foaming and emulsification processes needs to be considered, which includes aspects such as wettability of the channel walls, moving contact lines, and dynamic surface tension due to adsorption of emulsifiers. Incorporating these aspects introduces new challenges and a separation of length scales that increase the computational complexity and cost. A range of numerical simulations, and the associated challenges are reviewed in-depth by Wörner (2012) [35].

### 3.2. Spontaneous Systems

#### 3.2.1. Microchannel (MC) Systems

**Figure 4:**
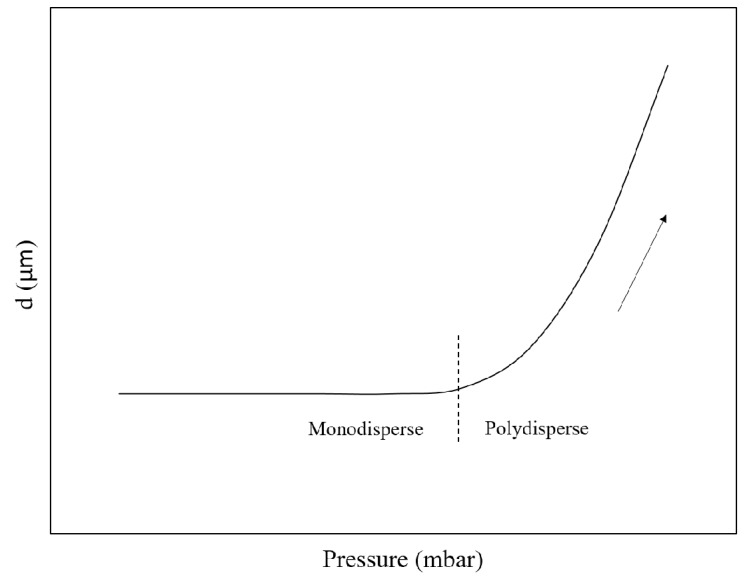
Schematic diagram showing the effect of dispersed phase pressure on the bubble (droplet) size distribution.

Spontaneous droplet formation is not significantly influenced by the continuous phase flow, in contrast to the shear-based droplet formation discussed above [36]. In a grooved MC system (see Figure 2d), Yasuno et al. (2004) [23] obtained foams with bubble size ranging from 33.6 to 51.1 µm and a coefficient of variation (CV) below 10%, and found that the bubble size was influenced by the continuous phase viscosity, yet independent of surface tension. Stoffel et al. (2012) [24] reported a two-stage bubble formation mechanism: The pressurized gas flows through a channel onto a shallow wide terrace, and then overflows into a deep channel (in which the continuous phase flow is present to carry away the generated bubbles). The radius of the curvature on the terrace gradually decreases, and when the critical radius is achieved a rapid pinch-off follows and a bubble is formed. The bubble size remains constant with increasing gas pressure in the so-called monodisperse regime, while as soon as the gas pressure exceeds a certain threshold (see Figure 4), the size distribution becomes polydisperse. This transition to polydispersity has also extensively been reported for emulsions, and has been defined by a critical capillary number (of the to-be-dispersed phase), which indicates that the viscous force becomes dominant over surface tension force [37].

**Figure 5:**
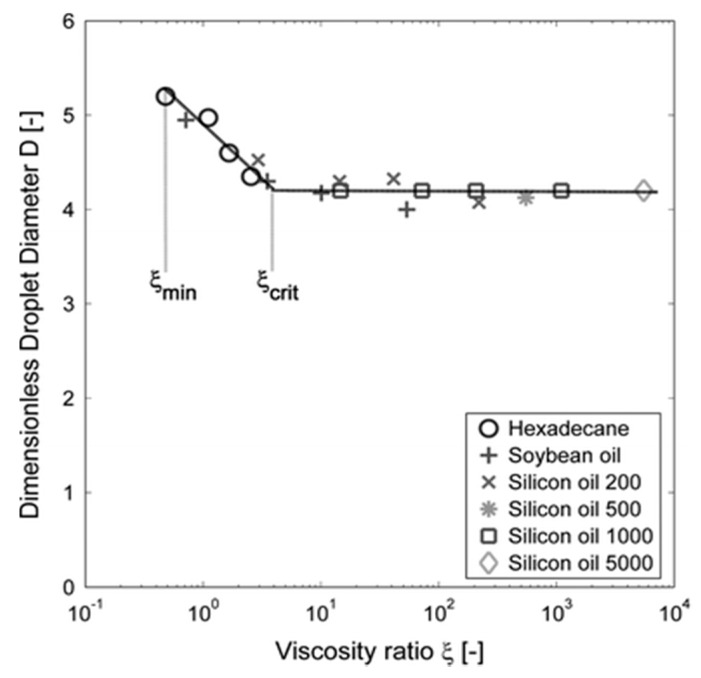
Plot of the dimensionless droplet diameter as a function of the viscosity ratio in terrace-based microchannels forming an oil-in-water emulsion. The viscosity of both phases is varied. Reprinted from van Dijke et al. (2010) [38].

**Figure 6:**
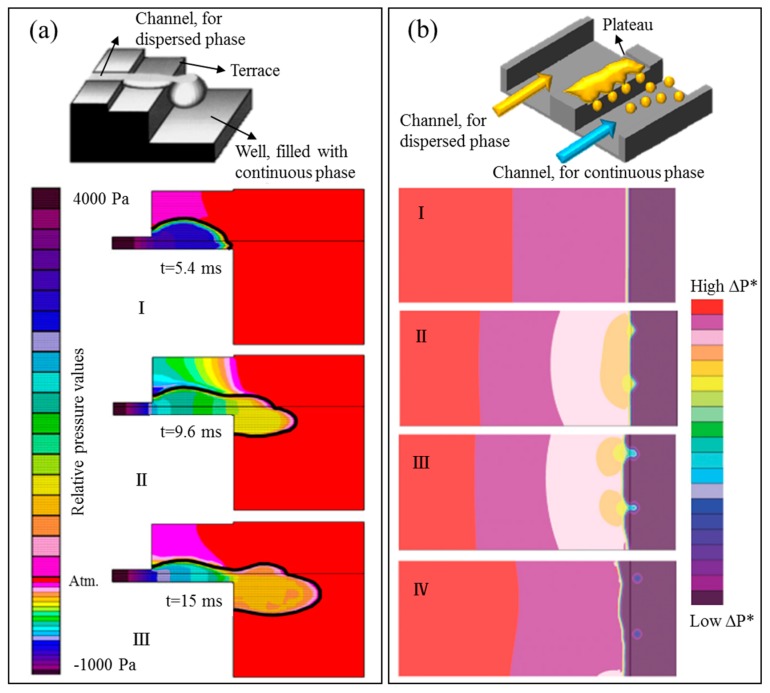
In (**a**), top: A forming droplet in the terrace-based MC system, adapted from Sugiura et al. (2002) [37] with permission from American Chemical Society; bottom: Half of the geometry of the MC system modeled to investigate the pressure gradient around the forming droplet at different time stages (I, II, and III) during emulsification, adapted from van Dijke et al. (2010) [38]. In (**b**), top: Droplet formation in an EDGE system, adapted from Sahin et al. (2016) [43]; bottom: Droplet formation on the plateau modeled from continuous phase filling the plateau, till the droplets are detached (I through IV), used to investigate the pressure gradient around forming droplets (light blue); adapted from van Dijke et al. (2010) [44] with permission from The Royal Society of Chemistry. In both systems, the pressure gradient near the droplet formation site becomes steeper with growing droplet size.

The viscosity ratio (*ξ*) between the to-be-dispersed and continuous phases is another important characteristic for MC systems. It has been extensively shown for emulsions that above a critical value of the viscosity ratio (*ξ_crit_*), the droplet size (within the stable pressure range) is constant. At lower viscosity ratios, the droplet size distribution is still monodisperse, but the droplet size is larger than that at high viscosity ratio (see Figure 5), which is linked to the slower inflow of the continuous phase onto the terrace due to higher viscous dissipation (see also Figure 6) [25]. When the viscosity ratio is below a minimal value (*ξ_min_*), the to-be-dispersed phase flows continuously from the terrace and droplet formation is impossible [38].

Given the much lower viscosity of air compared to oil (factor of 100), it was expected that bubble formation would not be possible with microchannel devices (based on Figure 5). However, monodisperse bubbles could be formed in a MC device [23], as well as in the EDGE devices that are discussed later [25]. This is likely due to the combination of low viscosity and compressibility of the gas phase (which is normally air), which induces a different formation behavior compared with emulsification in the same system. On the other hand, possibly again due to its low viscosity, the volume of air that could be dispersed was found to be a lot higher than the volume of liquid for emulsions; the bubbles being significantly larger than the droplets, typically by a factor of 3–4.

For the underlying formation mechanism, spontaneous formation as a result of minimization of surface energy (as found for emulsions) is also expected to be the case for foams. Rayner et al. (2004) [39] have studied this mechanism for emulsion formation using computational method (Surface Evolver), but to the best of our knowledge simulations for foam formation in microchannels have not been carried out yet. The predictive scaling of bubble size and modeling attempts of the foaming systems also still need to be performed, in contrast to the emulsification system.

Various geometries of microchannels have been developed, including straight-through and terrace-based types [26]. Geometry was identified to be a crucial factor determining droplet formation, in terms of both droplet size and operational stability. In the terrace-based MC system, for example, on relatively short or wide terraces, droplets (or bubbles) are formed instantaneously when the disk-like bulb reaches the edge of the terrace. In contrast, on a relatively long or narrow terrace, the bulbs continue growing when first moving into the deep channel. As a result, the latter formation process is less stable and the final droplet (bubble) size is larger [24,40]. An overview of spontaneous droplet formation devices is discussed in a review by Vladisavljević et al. (2012) [26]. In addition, Computational Fluid Dynamic (CFD) simulations were also used to define constraints for these devices [37,41]. The simulation demonstrated the existence of a pressure gradient in the continuous phase on the terrace of the microchannel (see Figure 6a). When this leads to reduced inflow of the continuous phase, larger albeit monodisperse droplets are formed. It was reported that the droplet diameter was typically 2.5–4 fold the height of the terrace in a grooved MC system [37]. Importantly, the aspect ratio between the long and short axis of the channel cross section should be sufficiently large to support successful droplet formation in a straight-through MC system. Channels with near-circular or square cross-section instead have a continuous outflow of dispersed phase. Numerical simulations have shown that the critical aspect ratio is 3–3.5 for channels with elliptical cross-section [41] and 2.6 for rectangular cross-section [42].

#### 3.2.2. EDGE

The main difference of EDGE (see Figure 2e) compared with microchannel devices is the much wider droplet formation plateau that connects the to-be-dispersed and continuous phases, and is capable of forming multiple droplets simultaneously. The plateaus are very shallow compared to the entry channel for the dispersed phase (see Figure 6b). This means that the highest hydrodynamic resistance is in the droplet formation unit itself, which ensures a steady plateau filling and enhances pressure stability compared to microchannel designs.

Numerical simulations of EDGE show that steep pressure gradients occur around the droplet formation locations (see Figure 6b), which can further explain the robustness of this system. As a result, the droplet size is constant over a relatively wide pressure range, and is 5.5–6.5 times the height of the plateau [44]. For foams, a first result shows that bubbles are at least 17 times the height of the EDGE plateau^19^, or 3–4 times larger than droplets. It is important to realize that the numerical simulations are very useful to predict trends, but do not cover all aspects that occur in practice. So far, they have been used to elucidate the effect of varying contact angles (which is a result of emulsifiers adsorption to the walls of the microfluidic devices) on the droplet size [45], or to predict the influence of different surface tensions [40]. However, the dynamic adsorption and redistribution behavior of emulsifiers at the interface (leading to a temporally changing surface tension) is still challenging to incorporate in the simulations due to the small length and time scales involved.

### 3.3. Up-Scaling Microfluidics

In order to make the production of food foams using microfluidics feasible, its productivity would need to be enhanced. The typical route towards up-scaling is to operate several formation units in parallel, as has been demonstrated mostly for emulsions [46,47,48], and limitedly for foams [49]. However, fabrication of such devices is currently far from trivial. An alternative approach is to use membranes that can be produced readily at lower costs, yet are less well defined in terms of pore geometries. Here we only mentioned membranes as an option to be complete and limit ourselves to upscaling of microfluidic devices.

The main issue that needs to be addressed for parallelization is the cross-talk between individual units. Cross-talk is known to significantly influence droplet detachment, in particular in parallelized shear-based devices. It is expected to be of even greater influence on bubble detachment, due to added complexity of compressibility and low viscosity of the gas phase [18,49,50] in comparison with emulsions. Shear conditions should be identical for all parallelized shear-based formation units to obtain monodisperse bubbles (or droplets). Thus, the units should be organized such that they do not shield others from the shear flow needed to detach bubbles, although this is difficult to realize.

The shear flow has a smaller influence in spontaneous devices, since the bubble size is determined predominantly by the to-be-dispersed phase, and remains constant within a certain pressure range (typically hundreds of mbar). Still, for spontaneous droplet formation in microchannels it has been reported that polydispersed droplets are formed as a result of relatively small pressure fluctuations [51]. The EDGE system is much less pressure sensitive, and shows a wider pressure range in which monodispersed droplets are produced [25,36]. When introducing partitions on the main plateau (the so-called partitioned-EDGE, see Figure 2f), this leads to well-defined droplet formation locations while further increasing the pressure stability, and thus to a hundred fold increase in emulsion productivity [52]. We expect this to be a promising route towards large-scale production of monodisperse emulsions (foams).

Efforts are continually put on these spontaneous devices for in-depth study, for instance, a similar geometry, which takes the advantages of both EDGE and MC systems (and buoyancy effects), was recently used by Opalski et al. (2019) [53] to study geometry effect on the throughput of monodisperse emulsion. The throughput of monodisperse emulsion in this geometry can be enhanced, and at the same time, a smaller increase in the volume of droplets was observed compared to a traditional MC system.

As mentioned previously, CFD can be used to simulate the droplet formation process at a single formation unit. Yet, there are much fewer simulations of multiple formation units that are working simultaneously. These larger-scale simulations would be an essential step to better understand how the parallelized droplet formation units work, including the relative influencing aspects. For example, Hoang et al. (2014) [54] developed a bubble-splitting distributor that contains 8 parallelized channels (including seven splitting distributors). Both their theoretical and experimental results indicate that the asymmetric flow due to fabrication inaccuracies and non-breaking bubbles lead to polydispersity, even if a bypass channel is used to better control the pressure.

### 3.4. Toward Food Grade Emulsifiers

In most microfluidic investigations low molecular weight surfactants such as sodium dodecyl sulfate (SDS) [23,25,55] were used as surface active components; only limited food-grade emulsifiers such as Tween 20 [13,18] have been reported. Maybe even more importantly, in many studies the dynamic effects that these surface active components can create, such as lowering of interfacial tension at the time scales of droplets (bubbles) formation are often overlooked.

In food products, proteins together with other components (e.g., polysaccharides) are the stabilizers of choice and they have an even more complex way of stabilizing interfaces. They have scarcely been used in combination with microfluidic devices. For example, Guell et al. (2017) [10] used a Y-junction to study the apparent interfacial tension of bovine serum albumin and whey protein-stabilized emulsions, Muijlwijk et al. (2017) [11] studied stability of protein-stabilized emulsions, and Dijke et al. (2010) [25] used proteins for emulsification and foaming with EDGE devices.

The limited use of proteins is most probably because in fundamental sciences within which microfluidics have been extensively tested, model systems with well-defined characteristics are preferred. Proteins have high complexity and are known to induce wettability changes [43], which is an undesired side-effect. To prevent the binding of proteins to the microchannel walls, and thus wettability changes in time, the walls need some form of modification, which may be as simple as a pre-adsorbed protein layer. Furthermore, the knowledge available for low molecular weight surfactants (e.g., the adsorption dynamics to the interface) needs to be translated to protein adsorptions (see analytical tools section). As mentioned before, proteins show much more complex adsorption behavior than well-studied low molecular weight surfactants, but luckily also for this microfluidic tools are available and have been used as described next.

## 4. Analytical Tools

### 4.1. Critical Parameters for Foam Formation and Stability

Common emulsification methods such as stirred tanks, colloid mills, and high-pressure homogenizers, operate under a wide range of flow regimes. Three common flow regimes are the laminar viscous (LV) at low flow-Reynolds number, turbulent viscous (TV) and turbulent inertial (TI) [56]. Table 2 shows that in all flow regimes the resulting droplet size depends crucially on interfacial tension. Yet its actual value during the droplet formation is very hard to determine, if at all. Only very recently, microfluidic platforms have been designed to measure dynamic interfacial tension for liquid/liquid systems under laminar flow conditions. The (sub)millisecond time scales that can be accessed in this way are not only relevant for emulsification within these microfluidic devices but also for large-scale operations, particularly those operating in the LV flow regime. Besides, locally large velocity gradient and the convection mass transfer mechanism were also indicated in microchannels [57]. Turbulent flow can be induced in microchannels, for example by adjusting the roughness of the channel wall [58]. Laminar flow transfers to turbulent flow at a Reynolds number of approximately 2000, and it occurs earlier with higher level of the roughness. Foaming experiments at high Re may help to produce more insights in large-scale operations. We discuss the dynamic measurement of surface tension below.

For the production of stable foams, two time scales are relevant: One relates to the formation of bubbles during which they should be protected against coalescence, and the other to life-time stability of the foam relating to gas transfer between bubbles. These time scales may be studied using microfluidic devices that were originally developed for emulsions. For example, a coalescence chamber has been suggested for early coalescence monitoring (see section below), and a micro centrifuge can be used for evaluation of stability under enhanced gravity. Although the latter one does not completely represent the situation of ageing products under normal gravity, the experiments yield useful information about long-term stability within manageable experimental time scales.

### 4.2. Interfacial Tension Measurement

In the scaling relations (see Table 2, Rayner and Dejmek, 2015) [56] used to predict droplet (and bubble) sizes, the interfacial tension *γ* and the related surface coverage *Γ* of emulsifiers are important parameters. Both parameters are only scarcely reported. Moreover, typically either the equilibrium interfacial (or surface) tension or that of an interface without emulsifiers is used, whereas in reality the interface will be partly covered and the actual interfacial tension is unknown since it changes dynamically. The typical time scales that are involved in bubble and droplet formation are in the (sub)millisecond range, and thus outside reach of conventional measuring methods such as the droplet volume tensiometer.

**Figure 7:**
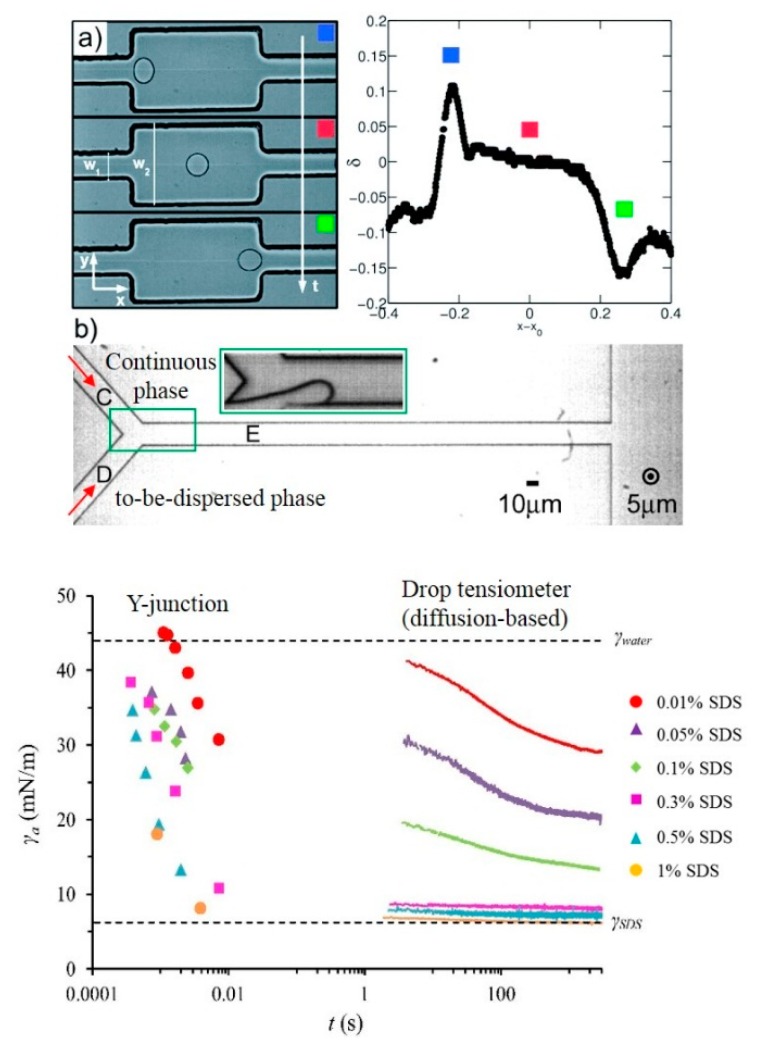
(**a**) Left: Diagram of the expansion cell; Right: The typical deformation (*δ*) of a droplet flowing in the expansion cell, reproduced from Brosseau et al. (2014) [62] by permission from the Royal Society of Chemistry. From the top (left) to the bottom (right), three important deformation points were indicated with different color squares: The droplet just enters the expansion, the elongated droplet relaxes back to a sphere, and it enters the next constriction. (**b**) Top: Layout of a microfluidic Y-junction with an angle of 97° between channel C and D [55], adapted with permission from the American Chemical Society; Bottom: The dynamic interfacial tension for hexadecane/SDS solution of different concentrations, derived by using Y-junction as a function of droplet formation time. As a comparison, the interfacial tension obtained with a droplet volume tensiometer is given on the right. Reprinted from Muijlwijk et al. (2016) [65], with permission from Elsevier.

In microfluidic devices, droplet formation can take as short as 1 ms (for bubbles this is even faster), so they provide a platform to study emulsification, and expectedly also foaming, at the length and time scales that are relevant for current large-scale processes. Microfluidic tools have already been used for characterization of liquid–liquid emulsification systems. For example, Brosseau et al. (2014) [62] used a microfluidic flow-focusing device to derive the dynamic surface tension from the deformation of a droplet before and after passing through a geometrical constriction (the expansion cell shown in Figure 7a). Wang et al. (2009) [63] determined the dynamic interfacial tension during emulsification using a microfluidic T-shaped device, and Steegmans et al. (2009) [55] using a Y-junction device (see Figure 7b). Güell et al. (2017) [10] further extended to using proteins, and compared their findings with premix membrane emulsification based on a modified Ohnesorge number* (Oh*). A lot of information was generated in our group using the Y-junction with which for instance, Muijlwijk et al. (2018) [64] carried out a comparative study on the dynamic adsorption behavior of different types of emulsifiers including food-grade ones.

Figure 7b shows an illustrative example of dynamic interfacial tension values determined with the Y-junction for a hexadecane/SDS solution system. The top horizontal dashed line denotes the interfacial tension between pure hexadecane and water, and the bottom one indicates the equilibrium interfacial tension in the presence of SDS. On the left, curves are shown for very fast droplet formation in the microfluidic Y-junction, with interfacial tensions derived from a calibration curve. In doing this, we were able to distinguish effects of varying emulsifier concentrations at specific droplet formation times. On the right, interfacial tension curves are shown generated with a droplet volume tensiometer that operates under diffusive conditions. It is immediately clear that the Y-junction can operate at much shorter time scales, which is due to enhanced mass transfer conditions. This is relevant for both microfluidics and other large-scale emulsification devices operating in the LV flow regime, while the drop volume tensiometer provides no access to these short time scales. In order to translate the technique to foams, it should be noted that bubble formation is even faster than droplet formation. Consequently, high-speed cameras are needed to record the fast bubble expansion.

### 4.3. Coalescence Behavior

**Figure 8:**
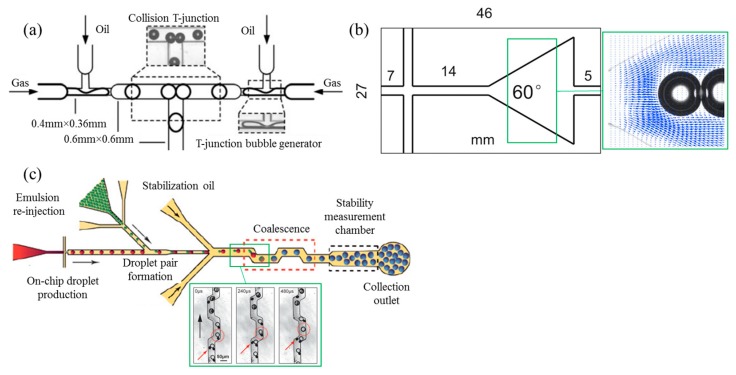
(**a**) Microfluidic system for 1-on-1 bubble interaction, reprinted from Yang et al. (2009) [66]. (**b**) Microfluidic expansion device and the velocity field (obtained by the micro Particle Image Velocimetry technique, micro-PIV) around the aligned bubbles, reprinted and adapted from Fu et al. (2015) [67] with permission from Elsevier. (**c**) Droplet fusion device, consisting of a droplet generation junction, a coalescence channel and a detection unit, for the assessment of coalescence stability; reproduced and adapted from Mazutis et al. (2012) [68] by permission from the Royal Society of Chemistry.

In less dense, polydisperse foams, both of the disproportionation and drainage processes contribute to the thinning and rupture of films between neighboring bubbles, and their subsequent coalescence (see Figure 1). Using microfluidics, specifically the film dynamics and bubble coalescence can be studied in a so-called coalescence cell. Here, monodisperse bubbles are brought in close proximity to capture their initial coalescence stability. For gas–liquid systems, a microfluidic collision T-junction (see Figure 8a) was designed by Yang et al. (2012) [66] to investigate the 1-on-1 coalescence stability of bubbles under confinement. Controllable coalescence in this case was realized for a range of continuous phase viscosities and flow rates of both phases. Fu et al. (2015) [67] employed a microfluidic expansion section (see Figure 8b) to quantitatively study the coalescence mechanism of bubbles in non-Newtonian fluids. Depending on the flow rate of both phases, two different coalescence mechanisms were found for the bubble trains: Mechanism I, coalescence of two different bubbles; and mechanism II, recoalescence of two bubbles that broke apart from the same ‘mother’ bubble. For liquid–liquid systems, droplet fusion also has been investigated using microfluidic devices. Mazutis and colleagues [68,69] developed a device (see Figure 8c) for chemical reactions and biological assays, in which droplets are used as micro-reactors, and the start and termination of a reaction can be precisely modulated by controlling the fusion of the droplets.

Away from rather isolated coalescence events (droplet pairs), multiple coalescence events were studied using a microfluidic coalescence chamber that was developed by Krebs et al. (2012) [70]. Basically, droplets are generated at a T-junction, and allowed to interact in a wider channel. Using this device for instance, the coalescence of SDS-stabilized droplets were monitored, and the effects of flow velocity, oil viscosity (to-be-dispersed phase), and oil volume fraction on coalescence stability were investigated, and also enhanced interfacial mobility (Marangoni effect) [71] could be detailed.

**Figure 9:**
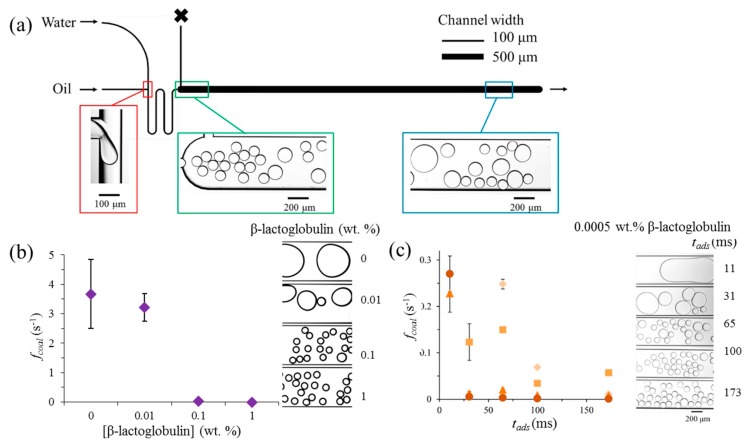
(**a**) Coalescence chamber, including a T-junction (red rectangle), a meandering adsorption channel, and a coalescence channel; reprinted from Muijlwijk et al. (2017) [11], with permission from Elsevier. (**b**) The coalescence frequency of bubbles as a function of protein concentration, at 100 ms adsorption time; reproduced from Muijlwijk (2017) [72]. (**c**) The coalescence frequency of droplets as function of adsorption time (achieved by varying the length of the meandering channel) and wt. % *β*-lactoglobulin (0.005, 0.001, 0.05, and 0.01, darker symbols correspond to higher concentrations); reprinted from Muijlwijk et al. (2017) [11], with permission from Elsevier.

Only very recently, Muijlwijk et al. (2017) [11] used this microfluidic coalescence chamber (see Figure 9a) to study the stability of protein-stabilized foams and emulsions, and took the technique one step closer to application for food products. The device consists of a T-junction where the two phases meet and either bubbles (Figure 9b) or droplets (Figure 9c) are made, an adsorption channel (the meandering channel, in which emulsifiers can further adsorb to the interface), and a coalescence chamber. In this way, the time scales for adsorption and coalescence could be decoupled. Coalescence frequency was derived from analyzing (image analysis) the droplet (bubble) size at the beginning and the end of the coalescence chamber (as illustrated in Figure 9a).

Although considerably less data is available for bubbles, the experiments shown in the PhD thesis of Muijlwijk (2017) [72] are a proof of principle that the coalescence chamber is also suited to monitor foams. Bubbles were stabilized with *β*-lactoglobulin solutions of different concentrations, and a clear dependency of coalescence frequency on concentration was found (see Figure 9b). A minimum *β*-lactoglobulin concentration of 0.1% was needed to keep the bubbles stable, which is approximately 20 times the amount needed for emulsions studied in the same device (which is discussed later). When keeping in mind that the bubble formation process is a lot faster than droplet formation, it is not that surprising that the bubbles had comparatively lower proteins coverage (see also Figure 7 for the effect of expansion rate), and thus lower stability than the much slower forming droplets.

Within investigations on emulsions, the layout of the coalescence chamber was varied, using different lengths of the meandering channel (Figure 9a), which allows for variation of adsorption time for emulsifiers prior to entering the coalescence chamber where the droplets interact. The coalescence frequency was a strong function of the *β*-lactoglobulin concentration, and besides, there was a clear effect of the adsorption time on it (Figure 9c). As expected, with the same protein concentration the longer the adsorption time the more stable the droplets were. In another investigation, even the oxidative state of the proteins was evaluated, and as was found in regular emulsions large differences in coalescence stability were noted [73].

### 4.4. Enhanced Stability Testing

Overall product stability and long-term effects are crucial for the shelf life of a food foam, but cannot be accessed by the coalescence chamber that operates at very short time scales after bubble formation. For polydisperse foams, the diffusion of gas from small to large bubbles (disproportionation) leads to coarsening [74]. This highlights the need for creating monodisperse foams. Although Xu et al. (2006) [15] reported that different types of emulsifiers can influence the interfacial viscoelasticity and thus potentially limit gas diffusion through e.g., protein-stabilized interfaces [23,75], it is not expected that this is a mayor influence due to the open nature of the protein layer.

**Figure 10:**
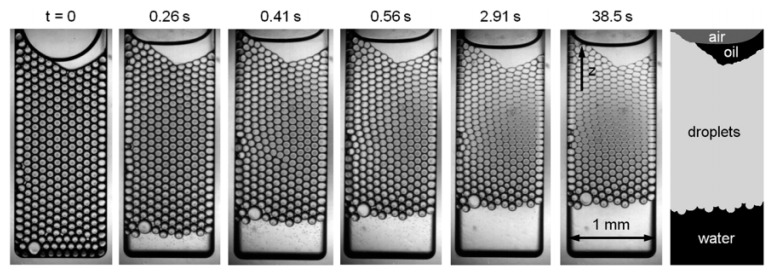
Images obtained with the micro centrifuge in which a small container filled with droplets is spun around at high g-force. The emulsion used in this experiment is stabilized by 10 mM SDS, and droplets have a diameter of 97 μm. Through image analysis, the amount of separated oil, aqueous phase and droplets, as well as droplet size was determined (marked in the last image). Reproduced from Krebs et al. (2013) [76] with permission from The Royal Society of Chemistry.

A deeper understanding of the ageing mechanisms and the corresponding critical conditions are relevant for effective control over bubble destabilization, and it is expected that microfluidics can make a contribution to this. The so-called micro centrifuge has been used for emulsion stability testing at high *g*-force (see Figure 10) [76]. An emulsion is first created in a separate microfluidic device and transferred into a small container-like microfluidic chip that is mounted onto a rotating device. The chip is spun in front of a carefully synchronized high-speed camera. From the obtained images, the size of the droplets, their deformation, and also coalescence behavior could be derived. Under enhanced g-force condition the (highly stable) droplets do deform greatly to a honeycomb structure, yet they do not coalesce (see Figure 10). For less stable emulsions, extensive coalescence was observed that could even be related to the critical disjoining pressure [77].

This analytical tools section indicates that many behaviors related to foam formation and stability can be investigated with microfluidic devices at relevant time scales. Although more research has been published for emulsions than foams, we believe that the developed devices can be easily extended to the study of bubbles. We foresee that this microfluidic toolbox could even be expected to become a good starting point for designing foam formulation using a bottom-up approach.

## 5. Toward Practical Application

Microstructures in novel foods (1–200 µm elements) [78] are in the range that can be made with microfluidic devices under very well controlled conditions. Foams are in the upper part of this range (20 to 200 μm) [79], for instance, the air bubbles in ice cream normally are around 20–50 μm [80]. It is known that small bubbles with controlled size distribution can modify the texture and appearance of food products by reducing the influence of buoyancy and vertical stratification, leading to creamier perception [81]. Through up-scaling of microfluidic devices, this can become a reality.

Disproportionation (which is caused by the polydispersity of bubbles) is the most important destabilization mechanism in food foams, because of the challenge to control size distribution of bubbles produced with conventional foaming techniques. Accordingly, solidification is often used to improve the stability of aerated food products that in principle can be made in rather monodisperse fashion by using microfluidic devices. The solidification method is, however, not relevant for all food foams, for instance cappuccino foam [82]. Thus using microfluidic devices for monodisperse food foams production is highly promising. The monodisperse foams, which are less prone to disproportionation, can also help to improve the sensory properties of aerated products, for example, beer foams.

The use of microfluidics in food foam and emulsion preparation (and investigation) may also lead to better use of the starting materials (e.g., emulsifiers). For example, currently excessive amount of emulsifiers is used in food production. Berton et al. (2011) [83] mentioned that during high-pressure homogenization an excess of at least 30% of *β*-lactoglobulin was needed (compared to monolayer coverage) to protect the emulsions against coalescence within 46–72 hours. In the microfluidic system, this amount can be reduced through controlled production leading to higher fraction of adsorbed proteins. For example, Muijlwijk et al. (2017) [11] indicated that only 0.005 wt.% *β*-lactoglobulin (which is close to the theoretical monolayer surface coverage) was sufficient for the emulsions to be stable in their experiment.

## 6. Conclusions—What Is the Future for Microfluidics and Food Foam?

Following the demonstrated value of microfluidics in the formation of droplets (emulsions) and, to a limited extent, bubbles, we foresee that this technique could have major contributions to the study and production of food foams. Here, we formulated four main potential applications:

1. Dynamic adsorption of proteins and novel stabilizers to the air–water interface. Since food foams are often stabilized by a mix of stabilizers, adsorption should be studied from the (sub)millisecond bubble formation time to long-term redistribution of components within the foam. In particular, the short time scale requires microfluidic tools similar to those used to measure interfacial tension of emulsion droplets—albeit at even shorter time scales. With current high-speed recording techniques these time scales can be easily accessed. This will allow us to unravel the adsorption mechanism of emulsifiers (proteins), and stabilization mechanisms of novel stabilizers such as food-grade rigid or soft particles, and aid in selection of suitable stabilizers for food foams.
2. Microfluidic tools to study bubble disproportionation. Disproportionation is the fastest destabilization mechanism of foams—in contrast to emulsions where the dispersed phase has low solubility in the continuous phase. The development of a microfluidic tool to study disproportionation can yield insights in the disproportionation dynamics of well-defined, bi-disperse foams. In addition, it could be used to select stabilizers that limit gas dissolution, and formulate a maximum degree of polydispersity that still allows for homogeneous food foam for the duration of the product’s shelf life.
3. Turbulent flow regimes of foam formation in a microfluidic device. It would be interesting to achieve turbulent conditions during foam formation in a microfluidic device (for example using wall roughness or by agitating the flow). This would yield insights in the different formation and stabilization mechanisms under the variety of flow conditions found in commercial foaming approaches.
4. Upscaling of microfluidic platforms for high-throughput food foam production. The key benefit of producing food foams in an up-scaled microfluidic platform is the bubble monodispersity that prevents disproportionation. At the same time, to assure this monodispersity, the bubble formation mechanism, stabilization, and cross-talk between pores should be well understood and tightly controlled. An interesting difference compared with emulsification is that bubble formation rates are extremely high, and should be balanced with a timely adsorption of emulsifiers, or with another stabilizing effect (e.g., using particles) to prevent early coalescence. Here, continuous phase flow and the gas/liquid buoyancy difference may aid in separating bubbles during their initial stabilization.

To conclude, microfluidics can play a two-fold role in understanding bubble formation and stabilization (point 1–3), as well as the large-scale production of monodisperse foams (point 4). This bottom-up approach provides a promising route towards novel food foams with superior properties, such as well-defined bubble size, structure homogeneity, added functional ingredients, and an enhanced shelf life.

## Figures and Tables

**Table 1:** Summary of dimensionless numbers and their definitions.

Name and Symbol	Definition	
	Equation	Ratio of Forces
Capillary number, Ca	$\frac{\eta v}{\gamma }$	$\frac{\mathrm{Viscous}\;\mathrm{stress}}{\mathrm{Interfacial}\;\mathrm{stress}}$
Weber number, We	$\frac{\rho v^{2}L}{\gamma }$	$\frac{\mathrm{Inertial}\;\mathrm{stress}}{\mathrm{Interfacial}\;\mathrm{stress}}$
Reynolds number, Re	$\frac{\rho \mathrm{vL}}{\eta }$	$\frac{\mathrm{Inertial}\;\mathrm{stress}}{\mathrm{Viscous}\;\mathrm{stress}}$
Ohnesorge number, Oh	$\frac{\eta }{\sqrt{\rho \;\gamma \;L}}$	$\frac{\mathrm{Viscous}\;\mathrm{stress}}{\sqrt{\mathrm{Interfacial}\times \mathrm{inertial}}}$

Symbols: η, viscosity (Pa·s); ρ, density (kg·m^−3^); v, velocity (m·s^−1^); γ, interfacial tension (J·m^2^); L, characteristic length (m).

**Table 2:** Summary of equations for estimating expected mean droplet diameter, stresses exerted on droplets, and relative time scales in emulsification process under laminar and turbulent flow conditions [56].

Flow Regime	Laminar-Viscous Shear or Elongational (LV)	Turbulent-Viscous Shear Forces (TV)	Turbulent-Inertial Forces (TI)
Re-flow	<1000	>approx. 2000	>approx. 2000
Re-droplet	<1	<1	>1 *
Mean Diameter (d≈) ^#^	$\frac{2{\;\gamma \;\mathrm{We}}_{\mathrm{cr}}}{\eta_{c}\;G}$	$\frac{\gamma }{\sqrt{{\varepsilon \;\eta }_{c}}}$	(γ3ε2 ρc)15
External stress acting on droplets (σ)	$\eta_{c}\;G$	$\sqrt{{\varepsilon \;\eta }_{c}}$	$\sqrt[{3}]{\varepsilon^{2}{\;d}^{2}{\;\rho }_{c}}$
Droplet deformation time scale ($\tau_{DEF})$	$\frac{\eta_{d}}{\eta_{c}\;G}$	$\frac{\eta_{d}}{\sqrt{{\varepsilon \;\eta }_{c}}}$	$\frac{\eta_{d}}{\sqrt[{3}]{\varepsilon^{2}{\;d}^{2}{\;\rho }_{c}}}$
Duration of disruptive stresses ($\tau_{DIS})$	$\frac{1}{G}$	$\sqrt{\frac{\eta_{c}}{\varepsilon }}$	12(γ2ρcε3)15
Surfactant adsorption time scale ($\tau_{ADS})$	$\frac{6\;\pi \;\Gamma }{{d\;m}_{c}\;G}$	$\frac{6\;\pi \;\Gamma }{{d\;m}_{c}\;}\;\sqrt{\frac{\eta_{c}}{\varepsilon }}$	$\frac{\Gamma }{m_{c}}\;\sqrt[{3}]{\frac{\rho_{c}}{d\;\varepsilon }}$
Droplet collision time scale ($\tau_{COL})$	$\frac{\pi }{8\;G\;\phi }$	—	$\frac{1}{15\;\phi }\;\sqrt[{3}]{\frac{d^{2}{\;\rho }_{c}}{\varepsilon }}$

Source: The data was adapted from Walstra, 1993 [59]; Walstra and Smulder, 1998 [60]; and McClements, 2005 [61]. Symbols: Re, Reynolds number, either on the scale of the flow or the droplet; d, droplet diameter (m); γ, interfacial tension (J·m^2^); We, Weber number; η_c_, viscosity (Pa·s); G, velocity gradient (s^−1^); ε, power density (Js·m^−3^); ρ, density (kg·m^−3^); Γ, surface excess of surfactant (mol·m^−2^); m_c_, surfactant concentration in the continuous phase (mol·m^−3^); Φ, volume fraction of the to-be-dispersed phase (m^3^ m^−3^); σ, stress (Pa); τ, characteristic time (s); subscripts: cr, critical value for droplet break-up; c, continuous phase; d, to-be-dispersed phase;.* For d > η_c_^2^/γρ; ^#^ Only if η_d_ >> η_c_.
